# *Bartonella* Endocarditis Mimicking Crescentic Glomerulonephritis with PR3-ANCA Positivity

**DOI:** 10.1155/2018/9607582

**Published:** 2018-08-19

**Authors:** Joseph Vercellone, Lisa Cohen, Saima Mansuri, Ping L. Zhang, Paul S. Kellerman

**Affiliations:** ^1^Department of Internal Medicine, Oakland University William Beaumont School of Medicine, Royal Oak, MI, USA; ^2^Department of Pathology, Oakland University William Beaumont School of Medicine, Royal Oak, MI, USA

## Abstract

*Bartonella henselae* is a fastidious organism that causes cat scratch disease, commonly associated with fever and lymphadenopathy but, in rare instances, also results in culture-negative infectious endocarditis. We describe a patient who presented with flank pain, splenic infarct, and acute kidney injury with an active urinary sediment, initially suspicious for vasculitis, which was subsequently diagnosed as* B. henselae* endocarditis.* Bartonella* endocarditis may present with a crescentic glomerulonephritis (GN) and elevated PR3-ANCA antibody titers, mimicking ANCA-associated GN, with 54 cases reported in the literature. Unique to our case in this series is a positive PR3-ANCA antibody despite a negative IIF-ANCA. Thus, the presentation of* Bartonella* can mimic ANCA-associated GN, and renal biopsy showing immune complex deposition is critical for diagnosis and appropriate treatment.

## 1. Introduction

*Bartonella henselae* is a fastidious organism commonly known for causing cat scratch disease. Cat scratch disease had been described over 50 years ago, but the first causal evidence of disease was not documented until 1983 [1]. Cat scratch disease typically presents with cutaneous lesions at the site of infection that progresses to lymphadenopathy and fever approximately two weeks after exposure to the bacteria. Visceral organ involvement, albeit unusual, typically involves the liver and spleen with marked hepato- and splenomegaly. Rarely,* B. henselae* results in culture-negative endocarditis, an illness that can be difficult to diagnose and a challenge to treat effectively and in a timely manner. Herein, we present a case of* B. henselae *with endocarditis, in a previous healthy male, causing crescentic glomerulonephritis with PR3-ANCA positivity mimicking an ANCA-associated vasculitis.

## 2. Case Report

A 47-year-old male with a past history of nephrolithiasis, irritable bowel syndrome, and mild depression presented to the emergency center with two weeks of flank pain and four days of cola-colored urine. He described a throbbing, stabbing pain in his left flank that persisted and progressively worsened, which was associated with dark urine, nausea, unmeasured fever, chills, and a 10-lb weight loss. He denied dysuria or urinary hesitancy.

On physical exam, vital signs showed a temperature of 37.2°C, blood pressure of 121/55 mmHg, pulse of 95 bpm, and respirations at 20 breaths per minute while saturating at 94% on room air. He was alert and oriented x 3, but in moderate distress from his left-sided flank pain. There was no cervical, axillary, or femoral lymphadenopathy present. On auscultation, he was noted to have bilateral, basilar crackles without rhonchi or wheezing. Cardiac exam showed a regular rate and rhythm, with a 2/6 systolic, crescendo-decrescendo murmur heard best over the left sternal border. There was severe, left CVA tenderness on exam, but his abdomen was soft, nondistended, and nontender. Extremities showed no edema, and skin exam showed no evidence of petechiae or rashes.

Initial laboratory data showed a WBC of 3.8 bil/L, Hgb of 7.7 g/dL, platelet count of 89 bil/L, sodium of 138 mmol/L, potassium of 4.4 mmol/L, chloride of 114 mmol/L, CO2 21 of  mmol/L, calcium of 7.4 mg/dL, phosphorus of 3.0 mg/dL, BUN of 19 mg/dL, creatinine of 2.36 mg/dL, and glucose of 97 mg/DL. Urinalysis showed 3+ blood, 1+ protein, > 50 RBC/HPF, 0-5 WBC/HPF, and RBC casts.

**Figure 1:**
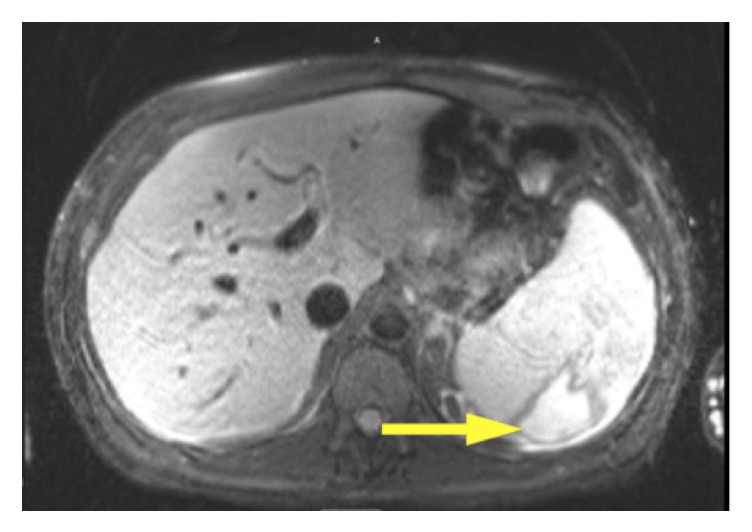
CT scan showing large splenic infarct.

Abdominal ultrasound showed a 12.6 cm right kidney, 12.4 cm left kidney with no hydronephrosis, and a spleen with wedge-shaped areas suggestive of infarct. An MRI showed splenomegaly of 17.9 cm and a wedge-shaped infarct (Figure 1)

Further blood test results showed a haptoglobin of 159 mg/DL, LDH of 272 U/L, fibrinogen of 248 mg/dL, an elevated CRP of 4.9 mg/dL, ESR of 25 mm/hr, C3 of 94 mg/dL, C4 of 23 mg/dL, negative antibodies to hepatitis A, B, and C, and negative ANA, ASO, and anticardiolipin antibodies. ANCA testing was negative using an indirect immune-fluorescent assay (IIF) with a positive lab test considered for results greater than 1:20. Myeloperoxidase antibody (MPO-ANCA) was negative, but proteinase-3 (PR3-ANCA) antibody titer was elevated at 160 units, using an enzyme-linked immunosorbent assay (ELISA) with a positive result greater than 21 units. Blood cultures were negative and remained so after 5 days.

**Figure 2:**
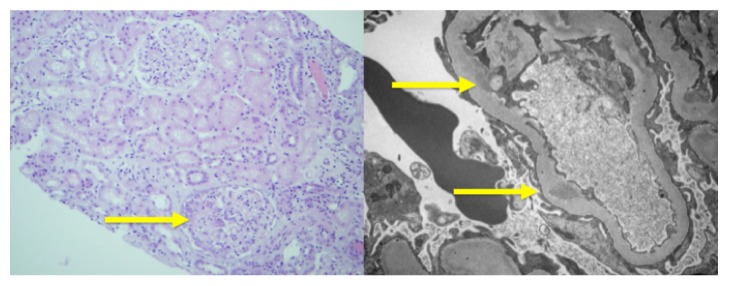
Light microscopy (left) of kidney showing focal proliferation with cellular crescent and electron microscopy (right) showing focal foot process fusion and subendothelial deposits.

A renal biopsy was performed. Light microscopy (Figure 2, left) showed focal proliferative injury with two nonnecrotic crescents. Immunofluorescence was positive for IgM, IgA, C3, and C1q located predominantly along the glomerular capillary loops and rarely in the mesangial areas. Electron microscopy (Figure 2, right) showed segmental foot process fusion with mesangial and subendothelial immune deposits with no subepithelial deposits, consistent with an immune complex GN.

Concerned with the heart murmur and renal biopsy results, a transthoracic echocardiogram was performed and was negative for valvular vegetations. A subsequent transesophageal echocardiogram showed a bicuspid aortic valve with a vegetation. Culture-negative endocarditis was diagnosed and valve replacement performed with pathology showing necrosis, neutrophils, and* B. henselae* on tissue culture and specialized stains.

The patient received 6 weeks of antibiotic therapy with doxycycline and rifampin and clinically improved with decrease in flank pain. Urinalysis also improved showing 4-10 RBC/HPF, 0-5 WBC/HPF, and no visible casts. Creatinine decreased to 1.4 mg/dL, and ESR and CRP normalized within 2 months to 3 mm/hr and <0.4 mg/dL respectively. Repeat proteinase-3 antibodies remained elevated at 121-163 units despite antibiotic therapy.

## 3. Discussion

Initial testing for ANCA-associated vasculitis typically uses IIF-ANCA. The specificity of ANCA testing is very high, with a very low false negative rate, but measurement of PR3-ANCA or MPO-ANCA antibodies with a positive IIF-ANCA improves sensitivity by ruling out false positive tests.

Positive tests for IIF-ANCA, PR3-ANCA, and MPO-ANCA antibodies may be found in patients with subacute bacterial endocarditis. Common organisms include Viridans streptococci, Staphylococcus aureus, and other staph species. The association of infectious endocarditis with these antibodies has led to postulated causal mechanisms for vasculitis. Unmethylated CpG is a constituent of bacterial DNA and has been shown to stimulate ANCA production in B cells of ANCA-associated vasculitis patients. Staph aureus tsst-1 superantigen nasal carriage carries a high rate of relapse in granulomatous polyangiitis patients. Diseases with barrier dysfunction to microbes, such as inflammatory bowel disease, show increased incidence of ANCA positivity. Neutrophil extracellular traps (NETs), which play a role in extracellular killing of microbes, may also release ANCA-associated antigens [2].

On the other hand, a retrospective review of patients with IIF-ANCA-negative, positive MPO-ANCA, or PR3-ANCA antibody testing such as that found in this case, showed that only 1 of 38 of these patients actually developed ANCA-associated vasculitis. There is evidence for cross-reactivity in the assays, as PR3-ANCA-positive antibodies have also been found in nonvasculitic inflammatory conditions such as rheumatoid arthritis, inflammatory bowel disease, and SLE [3]. Most relevant to our case, in contrast to ANCA-associated vasculitis, endocarditis-associated ANCAs typically show immune complex deposits in the kidney and resolution of kidney disease with treatment of the infection. Thus, although there is argument for bacterial endocarditis antigens being causal for renal vasculitis, current evidence favors ANCA antibody production as a nonpathologic result of bacterial endocarditis.

**Figure 3:**
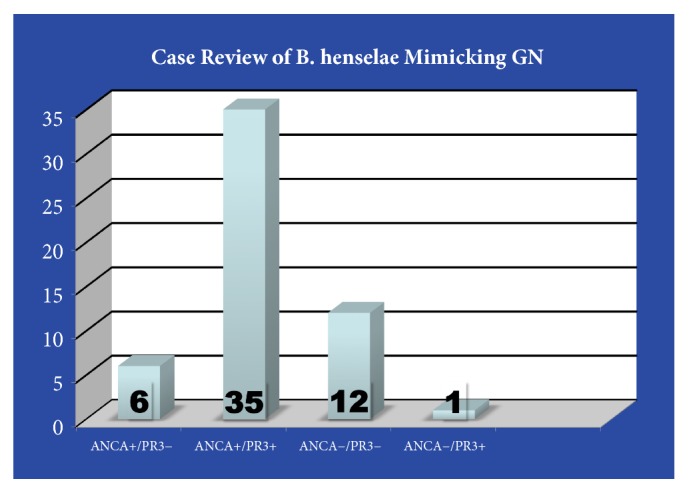
Case review [4–17].

We present a case of culture-negative endocarditis and acute kidney injury due to glomerulonephritis, due to* Bartonella henselae* cardiac valve infection. Culture-negative infectious endocarditis is estimated to comprise 3-48% of all endocarditis cases. A literature search revealed 54 cases of* Bartonella*-induced infective endocarditis associated with glomerulonephritis reported in 14 publications, with 77% of cases presenting with serologic positivity of either IIF-ANCA, PR3-ANCA, or both. Unique to our case is a high titer positive PR3-ANCA antibody with a negative IIF-ANCA (Figure 3). A review of glomerular light-microscopy findings associated with the aforementioned 54 cases of* Bartonella*-induced infective endocarditis demonstrated similar findings of focal proliferative injury with both necrotic and nonnecrotic crescents in both ANCA-positive and ANCA-negative cases [4–17]. Of the cases describing pathology in more detail, all but one showed positive immunofluorescence indicative of immune complex disease.

In summary, this case highlights how* Bartonella henselae* endocarditis may present with a crescentic and proliferative GN and elevated PR3-ANCA antibodies, thus mimicking an ANCA-associated GN. Because* Bartonella* is fastidious and often does not grow in blood cultures, as opposed to more typical endocarditis microbes such as Staphylococcus aureus and Viridans streptococci, clinical symptoms and lab results may lead to an incorrect diagnoses of ANCA vasculitis. An incorrect diagnosis may expose patients to immunosuppressive regimens potentially hazardous to patients with bacterial endocarditis. Thus, a kidney biopsy showing immune complex deposition is critical to establishing appropriate therapy.
